# Nothing about us, without us: is for us

**DOI:** 10.1186/s40900-022-00372-8

**Published:** 2022-08-04

**Authors:** Aamnah Rahman, Salma Nawaz, Eisha Khan, Shahid Islam

**Affiliations:** Born in Bradford – Bradford Institute for Health Research, Bradford, UK

**Keywords:** Public, Participation, Involvement, Engagement, Lay participation, 4MAT models and co-production

## Abstract

**Background:**

Public Participation Involvement Engagement (PPIE) is now strongly encouraged across health policy and research. Coproduction, although linked to PPIE is a way of working that can be applied to work collaboratively with participants in health. However, a lack of definition which leads to interchangeable terminology, limited guidance and examples of good practice on how to facilitate the process impedes progress. The Born in Bradford (BiB) research programme consists of a family of observational and longitudinal birth cohort studies (Raynor et al. in BMC Public Health 8:1–13, 2008; Dickerson et al. in BMC Public Health 16(1):1–14, 2016) which include participants from multi-ethnic and socially diverse backgrounds (Uphoff et al. in Int J Equity Health 12:1–12, 2013).

**Methods:**

This paper aims to highlight our approach to PPIE and coproduction methodologies, to provide an outline of the methods we have utilised to work collaboratively with our cohort populations from diverse communities and how we have managed to overcome challenges to achieve successful PPIE.A secondary aim of this paper is to demonstrate the value of PPIE and coproduction and how it can enhance research.  Some examples from recent years are provided to demonstrate how useful the approach has been for BiB community engagement and community participation. In addition, we discuss the methods we have used and how this methodology has now been embedded into protocol and practice in our research.

**Results:**

Successful and productive PPIE and coproduction occur where stakeholders are taken on board and realise the impact that their involvement can have in terms of policy design and delivery.

**Conclusions:**

The involvement of participants and the community in research about them becomes more credible when equal partnerships are formed and they are involved in the whole process leading to community ownership of research. Hence, nothing about us, without us—is for us.

## Plain English summary

Born in Bradford (BiB) is following the lives of more than 36,000 individuals to see why some families are healthier and others are not so. The children in the original study are aged range between 10 and 15 years, all born at the Bradford Royal Infirmary. Parents consented to take part in the study part prior to the birth of the child/ren (some families have more than one child in the study). BiB has been working successfully with grassroots communities for several years to increase community involvement in the research programme using various methods ranging from community engagement to coproduction.

“There has always been a balance, even earlier stages of the study when the focus was medical—it had to be plain (for the community). Everything we thought about did have an impact on BiB.”

The recruitment and establishment of proactive lay research groups is necessary to aid and shape our research. We believe that researchers need to ensure that they encourage community participation from all the various communities that make up the local population. Our approach serves a dual purpose as we want our communities to contribute to our research but at the same time, we want to offer local people skills development and confidence building so that they can articulate their views at various networks and events.

We wrote this paper to highlight some challenges, good practices and methods we have achieved to work with local communities.

## Background

Bradford is the sixth-largest metropolitan district in the United Kingdom with a population exceeding 530,000 with approximately 20% of the population living in the poorest areas of the UK [1]. Despite efforts to address health inequalities through policy and interventions, there are stark differences in people’s health depending on which part of the district they live in. There is almost a ten-year reduction in life expectancy for people who live in the inner city (dense over populated housing, excess pollution, poverty etc.) compared to an outer suburb (approximately ten miles apart) where people lead more affluent lifestyles. These gaps in health inequalities highlight the importance of targeted health interventions, particularly as current trends in light of the Covid-19 pandemic suggest the situation may now be worse. People from Black and Minority Ethnic backgrounds have been disproportionately affected by the pandemic [2].

The Born in Bradford research programme has been following the health and wellbeing of over 36,000 Bradford residents since 2007 [3, 4]. It hosts three birth cohort studies [1, 2, 5] (see Table 1) as well as an internationally recognised programme of applied health research with a focus on health inequalities in deprived and ethnic minority populations [5].

**Table 1:** The BiB research programme

Name of study	Recruitment years	Remit offer	Aims
Born in Bradford (BiB)	2007–2011	City of Bradford	Longitudinal birth cohort study
Born in Bradford Better Start (BiBBS)	2016–ongoing	Better Start Bradford areas	Experiential birth cohort Study
BiB Growing Up	2018–2021	Aimed at existing BiB cohort (8–11 years)	Extension of original BiB study
BiB4All	2019–2020	All pregnant women giving birth at Bradford Royal Infirmary	Routine data linkage of maternal care records
Age of Wonder	2022 onwards	Bradford District (14–21)	Adolescent years and post-18—emotional and social development

Public Participation Involvement Engagement (PPIE) is not a new concept; its origins in the UK go back as far as the Griffiths Report 1983. Public participation was enshrined into UK law by the NHS Community Care Act 1990 [6]. Successful engagement from all communities is important to ensure proactive representation in research for equality which has the community at its core, balanced opinion and assessment of the impact of involvement on different communities. Successful engagement and involvement lead to community ownership through coproduction methodologies.

PPIE is now widely accepted as important but gaps remain in the literature on how to do it well—we know the why but need to know the how-to [7]. One of the main issues is with the terminology used to define involvement and participation which adds to ambiguity [8, 9]. Often terminology is brandished to demonstrate inclusion in a positive manner but overlooks the key proponents of how they should be embedded into political philosophy and practice. The concept of coproduction was first introduced in health in the 1980s; it is implied when participants have contributed to the design of services [10]. PPIE and coproduction need to be integrally embedded into the core design, delivery, and implementation and monitoring of research. Therefore, appropriate resources and funding need to be adequately budgeted into any research proposal. 

“Research questions are used but our thinking is normal—we have said no when necessary so our advice was used.”

Whilst it is important and necessary for researchers to recruit proactive lay research groups to aid and shape our research to ensure research activities are relevant to the communities. A number of issues such as clarity about the purpose of applied health research impede BiB researchers’ efforts to engage and involve some communities appropriately, particularly those from White British working classes, marginalised groups within larger minorities i.e. newer arrivals from Eastern Europe to the United Kingdom and, asylum seekers and refugee groups. Many communities have reservations about involvement in research due to the current climate of hostility towards certain ethnic populations i.e. asylum seekers and refugees. Hence, there is fear, mistrust and suspicion about engaging with statutory agencies. The situation can be further exacerbated by the lack of sufficient English language skills in these communities. However, this is a minor limitation as it can be overcome with the use of interpreters or having researchers who speak community languages.

**Fig. 1:**
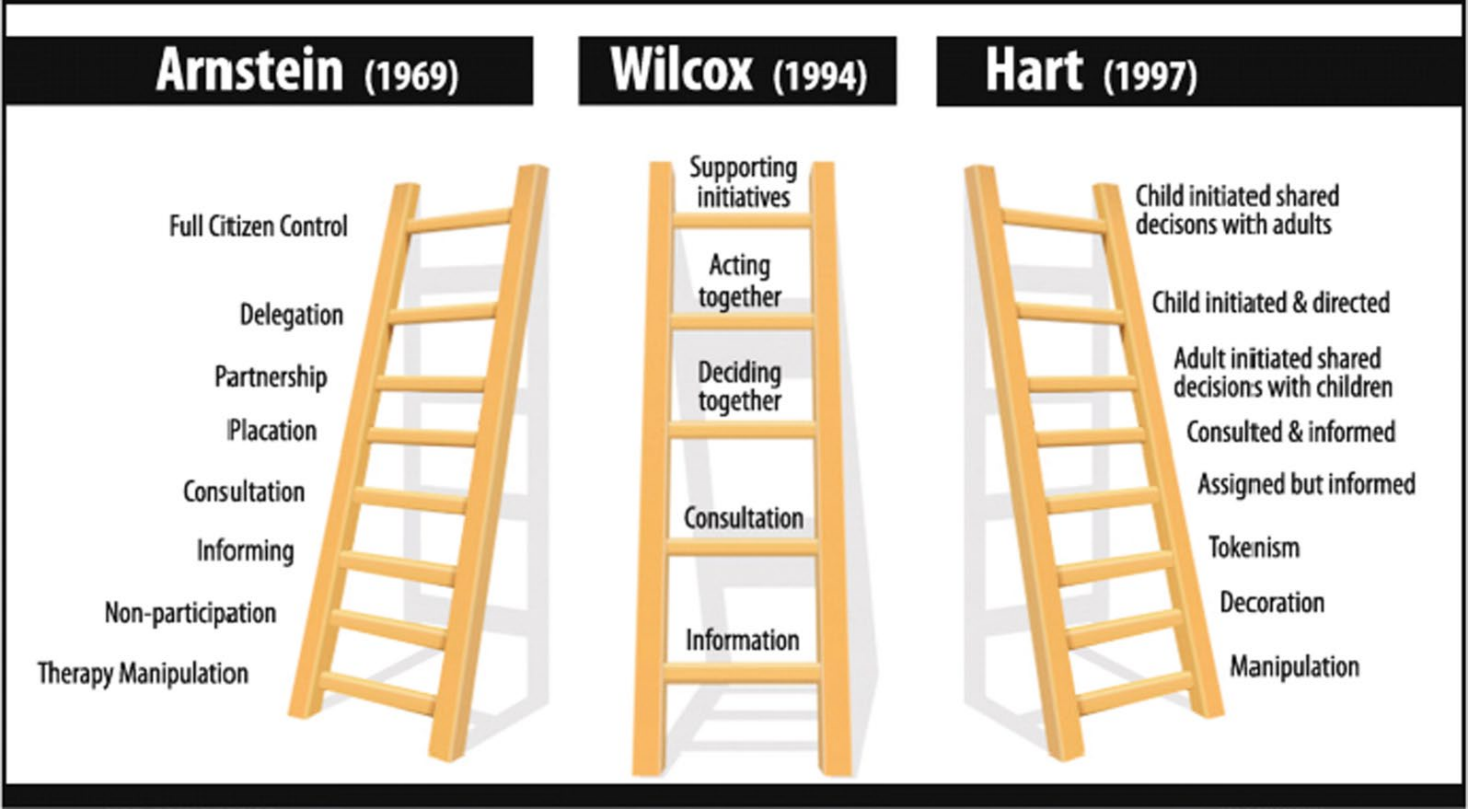
Ladders of participation [9]

The original model of participation by Arnstein [6] which was an oppositional model, showed that efforts in participation and contribution had been overpopulated by terminology that was exacerbated, rhetorical and often misleading. Wilcox’s model of participation [7] an adaptation of Arnstein’s model was developed to advance participatory research based upon collaboration. This model is based upon a ladder of participation to indicate participatory involvement in research ranging from providing relevant information, consultation, deciding together, acting together and supporting each other. It has been proposed that the Wilcox model is now outdated [8] and needs to be amended to take into account that participation itself is a goal for many people so PPIE needs to be established on rights-based relationships (equal partnerships) (Fig. 1).

However, as sceptics point out, this is not a straightforward process and there is a need to assess the impact of participation. There are calls for urgent evaluation on the impact of PPIE not only from the researcher's perspective but also from participants and their views on the impact of lay community participation. Examples of good research practice need to be highlighted where all parties feel their contribution had made a difference. BiB has attempted to do this through blogs about individual lay members and how they see their involvement has had an impact upon them as individuals, their families and the community. This will encourage researchers and participants to do more and also move the debate about public involvement in research forward.

“I was a dad who was the head of a children’s centre; it changed my attitude/learning and behaviour towards my third child. The other two were born before I knew all this stuff.”

## Methods

The National Institute for Health and Care Excellence (NICE) [10] guidelines on Community Engagement offers advice about local-based approaches to improve health and wellbeing that include building local assets. Similarly, national standards for public participation in research [11] state that research offers inclusive opportunities for people who are affected by and interested in the research at the earliest stage.

The BiB research programme is ‘people-powered’ (enabling communities to have a lead role in the type of research conducted in the city) and works closely with community members and stakeholders to coproduce and steer research priorities. BiB has been successful in building diverse and inclusive involvement and participation networks through a consistent and purposeful approach as much of our work is done with consultation with the cohort participants.

“BiB should use parents more. We feel our opinions are taken on board; it’s worth coming to these meetings. We feel our contributions are listened to.”

“Intrigued by the learning of researchers and difference in opinion, if BiB staff were asked what would be the one piece of advice that you would give to parents in Bradford?—All responses would be different even though the information was produced by BiB. Would love to be involved at the endpoint knowing there is a gap in how to work with parents. The research is done but how would you support parents to make a change?”

Similar to effective PPIE, coproduction increases interest and participation from the public in research. However, the term co-production is used frequently in research circles but it not fully understood so before we embark on co-production strategies we need to understand more about the definition of co-production that is being applied , is it coproduction or consultation? Are people being listened to, their experience and knowledge considered and their input acted upon? Is there a shared language being used between researchers and the community? A degree of flexibility and willingness to adapt are required to do it well [12]. Our findings show that having regular interaction and discussion with lay participation groups, improves discussion and allows researchers to focus on answering questions that communities want to understand. The appreciative enquiry [13] was developed to aid the design, implementation, monitoring and delivery of interventions for the community. BiB has utilised this staged model method to gauge interest from our communities about what is currently being researched, what is being done well and what could be done better? The final stage of this methodology will make the funders/commissioners and policymakers accountable to the communities of research. BiB has launched a coproduction strategy [14] as a result of this work and all further coproduction work will refer to this strategy.

Although more work needs to be done to address the issue of under-representation, BiB has utilised methods to link in with major organisations to have a presence at major community events in the city to disseminate research. BiB has formed strong partnerships with community organisations working with marginalised groups to communicate and involve them with our research. To facilitate community involvement in research, there is a need to convey to local communities why their participation is important.  On their part, communities will want to know what is in it for them and how they may benefit. As an example, to address these concerns, BiB has developed a leaflet for our communities from the Muslim faith to highlight that according to Islamic scriptures it is seen as an act of charity to get involved with research.

“I feel my English has improved, confidence improved. Before, the partner was not interested or supportive, thinking I’m just going for a meeting to be with friends but since reading a report he actually encourages me to attend, he prompts me to go. My children are much more disciplined as I’m learning things.”

The BiB research programme is active in the recruitment and development of lay research members to increase the involvement in research with communities in the Bradford District. The aim is to maintain a balanced and proportionate representation from all local communities. Community involvement is an ongoing part of our work and there are many lessons learned which have proved to be beneficial for both the participants and BiB. One of the methods BiB has utilised to increase involvement from the public is through setting up lay participation advisory groups. An initial lay research capacity group was set up in the early stages of the programme and subsequently, follow-up groups have been established. The BiB Parent Governors (PG) group was established in the early stages of the programme consisting of eight parents from various ethnic groups and socioeconomic classes in the BiB cohort. Most of the original people are still members of the group; the PGs are confident and articulate and can provide the BiB team with answers from a parental, professional and community perspective due to their links with communities and social networks.

The Community Research Advice Group (CRAG) consists of ten members from the Better Start Bradford (BSB) areas who are either a parent, work, live or volunteer in this community. The CRAG group was formed five years ago so that people from these communities could contribute to the discussion on prospective BiBBS research and its implementation, intervention and analysis. Some of the original members of this group have moved on due to gaining employment etc. and they have been replaced by new members. This is a positive development as it means people's involvement is reciprocated, they have been upskilled by developing transferable skills of confidence and articulation. More recently, BiB has established a group of Young Ambassadors (YAs). These are the oldest children in the original BIB cohort who have an interest in developing their skills to become lay advisory group members which will aid our research immensely in the coming years [15]. Additionally, community steering groups have been formed for specific projects within the BiB research programme including the Covid-19 BiB research project [16], the BiB breathes air quality clean air zone evaluation [17], Priority Setting Research [18] which is an adapted version of the James Lind (JLA) [19] approach to seeking community opinions on the top ten priorities BiB should focus on for children’s health and happiness. The Age of Wonder (AOW) which is the latest project following the social, emotional, mental and physical health of adolescents until they reach young adulthood has a community steering group which consists of community-based professionals and young people. Researchers from the AOW team work with young mental health apprenticeships, schools and other organisations to gauge community interest and coproduction for the future.

Impacts of working with lay participation groups such as PGs and CRAG include involvement with local, regional and national research programmes and initiatives. Examples include members participating in institutional research groups, NIHR collaborations and other academic institutions conducting similar research. Participants from these groups have also been involved with research from other academic units including The Health Experience Research Group (HERG) at Oxford University's Nuffield Department of Primary Care Health Science as case studies for lay research participation. More recently, they have been given training and paid to conduct qualitative research interviews with local communities using community languages for Covid-19 and pre-existing health conditions on behalf of University College London. In addition to gaining skills in qualitative interview methods, they have been given the opportunity to utilise their translation and transcribing skills.

Over the past decade, patients and families have been centrally placed at the epicentre of policy and practice [20]. However, huge obstacles remain in turning policy into practice. A point to note is that BiB does not class families taking part in the study as ‘patients’ which is the terminology used in most medical models of research, BiB involvement refers to the cohort as ‘participants’, ‘communities’ or ‘families’. Therefore, our PPIE and coproduction work is conducted with an objective mindset that envisages working with people in effective partnerships that provide a stable foundation for platforms to flourish in future endeavours.

In addition, the use of various theories of change logic models [21] such as the Public and Patient Engagement Evaluation Tool (PPEET) to assess involvement and participation has guided our approach to PPIE within BiB. PPEET tools have been adapted by BiB/BiBBS and produced into guides [22] designed to aid the design, implementation, monitoring and delivery of interventions for the community. The PPEET tool also encourages measuring the effects of PPIE through participants’ observation and reflection of their own performance [23].

The use of logic models ensures that the language is adapted and that input from the CRAG and PGs’ is taken aboard from community perspectives. If people feel they are being listened to and their input is valued, then they are more likely to engage further. For example, text messaging in addition to email is useful to keep participants updated (a lesson we have learned from BSB to reach wider audiences). Our study participants and lay research members from these communities have informed us that the text is the best way to reach them as they do not check their emails regularly.

Furthermore, to aid BiB’s communication with the public, each consultation with our PPIE groups is monitored for impact. BiB has embedded McCarthy’s 1987 model which combines Kolb’s 1985 learning styles 4MAT model [24] putting into practice the five ‘Ws’ of Why What, Who, When and Where to assess for impact and seek solutions for community-based initiatives. The model’s methods state that 4MAT is an 8-step order (connect, attend, imagine, inform, practice, extend, refine and perform) which is instructional in using Kolb’s four quadrants using the different sides of the brain (left and right) [25].

Community participation in research works well when the appropriate approaches and relevant engagement methods are utilised to reach the potential of communities. For example, BiB’s ActEarly researchers conducted an exercise using a community cookbook approach [26] around healthy living and food choices in a local area. Various stakeholders ranging from the local community, police, elected councillors, and local school teachers were invited to have their input during the meal. Whilst the original research was intended to be focused on food choices and buying power it became apparent that the communities’ focus was the social environment and safety which had a direct impact upon their food choices and buying power. Limited safe transport links to places where healthy and adequately priced food choices were available meant they were restricted to buying unhealthy and overpriced food in their local environment.

Skilled Data scientists and the community worked together and were able to have a change of territory in the shape of the questions and how they were being asked to  be more focussed and impact on the matter. Having  staff members that are relevantly skilled and linked to grassroots communities, ensures trust and rapport are established to conduct robust research. However, there are limitations as community engagement and participation require a constant impetus which most research programmes do not factor into their budgets. This sort of work is time-consuming and often requires networks to be built over a period of time. This conflicts with how research projects work—often quick-paced and require immediate results. Therefore, further consideration needs to be given to engagement/participation methodologies when planning research projects to ensure the expectations of the participants and researchers are met and managed appropriately.

Behavioural Change (amongst policymakers and the community) requires a commitment to act and implement the findings of the research, especially when they are of benefit to communities. Therefore, it requires the researcher(s) to have stakeholders in mind to ensure that they are kept informed and updated. Methods of current engagement need to be addressed and new methods need to be introduced. In the early days of the first lockdown (March 2020), BiB utilised its researchers to gauge the effect of the Covid-19 pandemic upon the local communities through seeking community soft intelligence [16] from the communities. This was communicated back to policymakers and commissioners to feed into the district strategy to manage the response to the pandemic.

## Results

BiB has developed an approach to community engagement to offer input on research strategy and routes to implementation, and extensive activities to keep cohort members informed and involved. Although these soft skills are not inherent in all researchers—they can be learnt and developed by working with grassroots communities. This is beneficial because it implies that this encompasses a process that is conducted purposefully and reflects research practice and, provides estimations of the effectiveness of co-production practices. This approach to PPIE has worked well for both the group members and the researchers. The other main reason why BiB has been successful is the process of identifying community readiness for the initiatives we wish to introduce, for example, community readiness assessment with the Roma community [27]. Alongside community readiness, the models of co-production and community capital have been addressed to harness community participation potential.

The BiB research programme has had to adapt to the challenges and it has resulted in the community engagement procedures being designed to factor in alternative methods of engagement such as working with existing organisations and networks. This is beneficial to us as these organisations and networks have established relationships with these communities which allow us to overcome the initial barriers of building trust and rapport. Effective community engagement is a crucial part of our work to ensure we overcome some of the challenges. Including communities in our research is an important factor, so BiB has invested in time and funding for lay participation groups. It has become a two-way process where we have achieved our aim to ensure we avail opportunities to engage our communities so that their opinions are taken into consideration in meaningful discussion. In turn, BiB receives and acts upon the feedback concerning our research from our local communities.

In practice at BiB’s PPIE meetings, researchers are invited to speak about their research proposal/project which results in being questioned in a format by encouraging questions and ideas from our lay involvement and participation groups. Thus taking involvement to a higher level, where coproduction techniques develop. In return, our lay research members have developed skills and confidence to a point where they can convey their viewpoint to academics, policymakers, funders and research platforms. BiB endeavours to ensure that we have community participation and involvement in our research from all the various communities that make up our local population.

BiB has tasked itself to become an organisation that has people-powered research at the forefront of its objectives so having the communities on board from the outset has been a fundamental part of the research process. The usefulness of this approach (involving research participants in engagement) includes outcomes showing that participation is advantageous to organisations and community representatives, particularly those from underprivileged communities [28].

It has been suggested that at the core of successful PPIE is the dynamic interaction of different forms of knowledge, notably lay and professional which can be tested against the theoretical framework [29]. BiB’s community representation brings unique skills and attributes to the research such as being participants in research, parents of children in the cohort with professional backgrounds and affiliates of various community and social networks. Our lay members often aid researchers to understand how communities will interpret the research and the impact it is likely to have.

BiB has established a Communications group that consists of researchers, administrative staff, lay members and senior managers to plan a coordinated communications strategy that should make messaging for BiB research consistent, timely and appropriate. Lay members can advise BiB research on how the community will perceive our communication.

“Newsletter and social media language and tone need to change a bit. Currently, the newsletter says the research found x, y and z (facts and statistics). It needs to say ‘would you like to be a part of a group to try out…’ and the report back.”

“Facebook—could have something on portion sizes. Not a packet of crisps but 3–4 crisps (allow for children’s stomach sizes). Often written in a judgemental tone i.e. what have you tried that works? Could be ‘Did you know we found this? You could do… Interesting tips section too could work really well on Instagram.”

Successful PPIE and its benefits across the BiB research programme has resulted in further developments of collaborative working across the Bradford Institute for Health Research on PPIE. BiB researchers and others working at Bradford Institute for Health Research have developed ideas further which include:

- Mentorship offers for lay participants will benefit the development, upskilling and moving forward of lay research participants. It will also create space for new people to come in and participate in research.
- Our Citizen Science approach encourages local communities to get involved in community research such as the environment and green spaces. The approach harnesses local talents to build local research capacity, especially amongst younger people. It will enable communities to actively approach and take ownership of local research and, the initiatives that are implemented as a result of the applied research.
- Volunteer roles/student placements and paid internships have been developed in BiB over the past few years. It is hoped that these will be expanded further as they enable people to learn about the research, and get to know the workings of the programme and research. It is hoped that those who take up these opportunities will come back to work for the programme, especially the younger members.
- Research Apprenticeships for local people who have an undergraduate degree and want to embark on a research career.
- Dissemination research—social media/WhatsApp messages etc. —appropriate, easy language and the appropriate platform for all our communities i.e. what does ‘people-powered’ mean? BiB has defined it as enabling communities to have a lead role in the type of research done in the city. But what do our communities take from this and does it have the same meaning to the public as it does to us.
- Signposting—BiB has developed a directory of local information and services including help for mental health issues which our participants can access (BiB was commended by the Health Foundation for doing this). This arose as a result of the Covid-19 research, where participants informed BiB that they did not where to access help from health and social services
- Development of a Centre for Peer-Led Research—a pool of community researchers will be trained for research involving local communities

## Discussion

Whilst there is overwhelming support in favour of PPIE style activities [28], limitations on guidance and researcher skills make the task difficult on how best to facilitate PPIE groups effectively and productively [29]. Added to this, there is a lack of dissemination of good practice [30]. Furthermore, information and dissemination and PPIE work with children are even more limited [31]. Dissemination is often overlooked or given less priority which erodes community interest and trust so BiB has established this as a central feature of PPIE.

“Feedback from students that I spoke about findings on physical activity. The student said, so what? Eh? So what? They do this research and then what happens next? Showing findings are still not communicated and there are levels of dissemination’’.

A systematic review in 2015 [30] which included 131 results showed there is sound evidence that community engagement interventions have a positive impact on health behaviours, health consequences, self-efficacy and perceived social outcomes. But it needs to be questioned as to whether it applies to research, particularly for lay capacity advisory groups and co-production approaches. Proponents promote community engagement as being more democratic and inclusive as such inclusion helps produce higher-quality research. Key features that should be considered are less likely to be omitted when people from within the community are included.

However, there is resistance from academics and researchers who are less in favour of co-production, they argue that is an idealistic view and not grounded in evidence. There are challenges in traditional academic practices and researchers who are not prepared to be flexible view it as risky, time–consuming, complicated, emotionally draining, lacking stability, potentially open to unexpected external events and also putting researchers in competition with others [28]. It is argued that the challenges include the relevance of the policies to the engagement and involvement work and they often lack diversity. Furthermore, the alignment of research priorities with those of the community is a causal factor in the lack of participation from communities in citizen science research [31]. BiB has been fortunate to engage a diverse range of members who are involved with our community participation and engagement. Also, such individuals’ experience of ‘their experience’ is instrumental for understanding how they will interpret and receive engagement.

As BiB’s lay participation groups have grown from the initial group formation and development through support and development of skills. Researchers support and encourage lay participants to contribute in a way that enables them to influence research plans that make a difference to the community. This has also had an impact on the knowledge base of the research team and other professionals. Sharing experiences from personal and community reflections have helped researchers understand the viewpoint from a community perspective which has shaped their practice in terms of design, delivery and implementation of applied health research.

“Findings from BiB helped to educate staff working within childcare and other related professions. It was far-reaching and real to here in Bradford.”

One of the elements in PPIE and Coproduction is reciprocity, what can researchers give back to the community. It has been engrained in BiB’s PPIE and Coproduction work. Lay research members have benefitted in that their confidence has grown, they can articulate and express their opinions to professionals and other organisations. It empowers such individuals further to know that their ideas, opinions and views are being listened to and taken on board. Alongside parenting skills, some have developed research skills through experience in community-based research which they can utilise in other capacities. Having members of our local communities with such skills is a source of pride for the programme as we have confidence in them to represent BiB.

“For ourselves, we have learnt and have had behaviour change i.e. turning car engine off around schools (BiB findings)”

“Data from children feed into the medical system. Well, that was good but what’s happening to my child? G.Ps the pass of information as BiB moved forward, individually it helped supporting our children.”

Although there are some disadvantages to this type of engagement, as asking a selection of people (albeit well connected and familiar with community issues) to comment and represent a whole community may not be the most appropriate approach. However, each person’s view is different depending on their experience, education, knowledge and perception. In addition, the numbers of people in attendance can vary so can potentially cause issues when the groups are asked to decide upon recommendations for various research projects. A minimal limit of two people in attendance at a meeting is set for it to be a quorum.

Similarly whilst coproduction is widely encouraged it is argued that only the positive aspects of co-production are highlighted [32], however, there are challenges too from the point of deciding on terminology and definition of co-production, to issues on its purpose, ultimate goals and methodology. Furthermore, it does not come free—all such factors come with risks (personal to public, researchers and wider cause) and price (effects of the process on research, researchers and stakeholders and academia/scholarship). Therefore, it is necessary to start with a clear purpose and strategy to understand who will be affected. Then select strategies and working outcomes that will best achieve the desired goals for the research and be beneficial for evaluation. A strong base for the evidence on the impact of co-production would enhance the research by adding credibility but a lack of such co-production would be disadvantageous because funders and policymakers may increasingly seek evidence to support that coproduction has been carried out.

Achieving co-production can be a slow, resourceful, delicate process and considering the above arguments, it can be a complex method. One of the reasons BiB has been successful in engaging communities from diverse backgrounds and age ranges is due to having researchers who are familiar with these communities and possess a skills base needed to work with communities. Developing trust and building relationships are criteria that are often essential to engagement but take time so it helps to have researchers who are experienced in this field. Sometimes community input in research is required quickly, so can only be done with long-established relationships for example, further to the initial community intelligence gathering work in the early phases of the Covid-19 pandemic [16] whereby BiB researchers were able to gather sensitive and pertinent data about the impact of the first lockdown from community members. Additional work was carried out by researchers alongside other health professionals and community activists around Covid-19 vaccine hesitancy and challenging misinformation around them [33].

There are calls for researchers to use their ‘soft skills’ (communication and experience working with communities) to provide participants with platforms where their knowledge and skills are heard and developed [31]. Disparities exist because the importance of involving the public in qualitative research by using a person-centred approach is widely acknowledged and appreciated but there is confusion as researchers do not always use person-centred and PPIE simultaneously, and often there is more focus on one approach [34].

## Conclusions

Whilst health research is encouraged to do PPIE work, there is a need for some guidance on how to do this effectively and proficiently, hence our decision to write this paper. There is a need to recognise that the ‘one size fits all’ approach is not the best approach. Every community and community of researchers have their unique challenges, talents, skill sets and resources—it is up to the researchers and lay participation groups to work on the aims and goals and, the strategy they are going to use to achieve these. Having clear and concise guidelines with models of practise to adhere to is advantageous, especially to those who are not familiar with the incorporation of lay participation in research. This model of working takes time, commitment and resources from both communities and researchers, but if done effectively can immensely increase the quality and relevance of research as well as impact positively on the communities in which we live. Therefore, it is necessary for PPIE and Coproduction to be a core part of any funding bid or grant and research projects need adequate resource and staffing to carry it out as a central element of the research. 

## Data Availability

Not applicable.

## Abbreviations

BiB: Born in Bradford
BiBBS: Born in Bradford Better Start
AOW: Age of Wonder
PPIE: Public Participation Involvement Engagement
PG: Parent Governors
CRAG: Community Research Advisory Group
BSB: Better Start Bradford
YAs: Young Ambassadors
NICE: National Institute Clinical Excellence
UK and NIHR: National Institute Health Research, UK
